# Expression profiles of NOD-like receptors and regulation of NLRP3 inflammasome activation in *Toxoplasma gondii*-infected human small intestinal epithelial cells

**DOI:** 10.1186/s13071-021-04666-w

**Published:** 2021-03-12

**Authors:** Jia-Qi Chu, Fei Fei Gao, Weiyun Wu, Chunchao Li, Zhaobin Pan, Jinhui Sun, Hao Wang, Cong Huang, Sang Hyuk Lee, Juan-Hua Quan, Young-Ha Lee

**Affiliations:** 1 Stem Cell Research and Cellular Therapy Center, Affiliated Hospital of Guangdong Medical University, Zhanjiang, 524001 Guangdong Province China; 2 Brain Korea 21 FOUR Project for Medical Science, Chungnam National University, Daejeon, 35015 Republic of Korea; 3 Department of Medical Science, Chungnam National University, Daejeon, 35015 Republic of Korea; 4 Department of Gastroenterology, Affiliated Hospital of Guangdong Medical University, Zhanjiang, Guangdong Province 524001 People’s Republic of China; 5 Department of Dermatology, Skin Research Institute of Peking University Shenzhen Hospital, Peking University Shenzhen Hospital, Shenzhen, 518036 Guangdong Province China; 6 Department of Internal Medicine, Sun General Hospital, Daejeon, 34084 Republic of Korea; 7 Department of Infection Biology, Department of Medical Science, Chungnam National University College of Medicine, 6 Munhwa-dong, Jung-gu, Daejeon, 35015 Korea

**Keywords:** *Toxoplasma gondii*, Human small intestinal epithelial cells, NOD-like receptors, Inflammasome, Caspase-cleaved interleukins

## Abstract

**Background:**

*Toxoplasma gondii* is a parasite that primarily infects through the oral route. Nucleotide-binding oligomerization domain (NOD)-like receptors (NLRs) play crucial roles in the immune responses generated during parasitic infection and also drive the inflammatory response against invading parasites. However, little is known about the regulation of NLRs and inflammasome activation in *T. gondii*-infected human small intestinal epithelial (FHs 74 Int) cells.

**Methods:**

FHs 74 Int cells infected with *T. gondii* were subsequently evaluated for morphological changes, cytotoxicity, expression profiles of NLRs, inflammasome components, caspase-cleaved interleukins (ILs), and the mechanisms of NLRP3 and NLRP6 inflammasome activation. Immunocytochemistry, lactate dehydrogenase assay, reverse transcription polymerase chain reaction (RT-PCR), real-time quantitative RT-PCR, and western blotting techniques were utilized for analysis.

**Results:**

Under normal and *T. gondii*-infected conditions, members of the NLRs, inflammasome components and caspase-cleaved ILs were expressed in the FHs Int 74 cells, except for NLRC3, NLRP5, and NLRP9. Among the NLRs, mRNA expression of *NOD2*, *NLRP3*, *NLRP6*, and *NAIP1* was significantly increased in *T. gondii*-infected cells, whereas that of *NLRP2*, *NLRP7*, and *CIITA* mRNAs decreased significantly in a time-dependent manner. In addition, *T. gondii* infection induced NLRP3, NLRP6 and NLRC4 inflammasome activation and production of IL-1β, IL-18, and IL-33 in FHs 74 Int cells. *T. gondii*-induced NLRP3 inflammasome activation was strongly associated with the phosphorylation of p38 MAPK; however, JNK1/2 had a weak effect. NLRP6 inflammasome activation was not related to the MAPK pathway in FHs 74 Int cells.

**Conclusions:**

This study highlighted the expression profiles of NLRs and unraveled the underlying mechanisms of NLRP3 inflammasome activation in *T. gondii*-infected FHs 74 Int cells. These findings may contribute to understanding of the mucosal and innate immune responses induced by the NLRs and inflammasomes during *T. gondii* infection in FHs 74 Int cells.

## Background

*Toxoplasma gondii* is an obligate intracellular protozoan parasite that infects one-third of the world’s population [1]. Infection is most commonly acquired through the ingestion of raw or undercooked meat containing the cystic bradyzoite form of *T*. *gondii* or through the ingestion of materials contaminated with cat feces that may contain *T*. *gondii* oocysts. Once inside the body, the parasite breaches the intestinal epithelial barrier and spreads from the lamina propria to other organs [2]. Intestinal epithelial cells can sense and respond to the invading microbial stimuli to reinforce their barrier function. They also participate in the coordination of appropriate immune responses [3]. The innate immune system plays a significant role in sensing pathogens and triggering biological mechanisms to control infection and eliminate pathogens [4, 5]. It is activated when pattern recognition receptor proteins, such as Toll-like receptors (TLRs) or nucleotide-binding oligomerization domain (NOD)-like receptors (NLRs), detect the presence of pathogens, their products, or the danger signals [5–7].

NLRs are a large group of cytosolic sensors that have diverse functions in innate immunity and inflammation. Based on the type of N-terminal domain, NLRs are classified into four subfamilies, NLRA, NLRB, NLRC, and NLRP, and an additional subfamily, NLRX1 [7, 8]. Several NLR molecules remain associated with the *T. gondii-*infection mediated immune responses in the infected hosts. It has been reported that *NOD2*-deficient mice are unable to clear *T. gondii* and fail to induce an appropriate adaptive immune response [9]. In addition to NOD2, NLRP1b and NLRP3 are also involved in rendering protection against *T. gondii* infection [10, 11]. In human acute monocytic leukemia cell line macrophages, the messenger RNA (mRNA) levels of *NLRC4*, *NLRP6*, *NLRP8*, *NLRP13*, *AIM2*, and *NAIP* are significantly elevated because of *T*. *gondii* infection, in a time-dependent manner [12]. Although some studies involving mice or cell lines have reported the involvement of NLR members in *T. gondii* infection protection [9–12], little information is available about the regulation of NLR activation in gut epithelial cells.

Ligand recognition by the NLR family members, such as NLRP1, NLRP3, NLRP6, NLRP12, and NLRC4, leads to the activation of inflammasome, a multiprotein complex, which cleaves interleukin (IL)-1β, IL-18, IL-33, and IL-37 (IL-17A) by caspases, the effector components of inflammasomes [8, 10–14]. *T. gondii* infection in cells with *NLRP1* knockdown fails to induce the production of inflammatory cytokines including IL-1β, IL-18, and IL-12 compared to control cells [10]. The broad range of pathogens that act on NLRP3 in several kinds of epithelial cells include *Plasmodium* sp., *Trypanosoma cruzi*, *Leishmania* sp., and *T. gondii* [15]. The P2X7R/NLRP3 pathway plays an important role in IL-1β secretion and inhibition of *T. gondii* proliferation in small intestinal epithelial cells [16]. While reports have revealed NLR activation by *T. gondii* infection in various cells, information on inflammasome activation in gut epithelial cells infected with *T. gondii* is very scarce.

NLRs play a crucial role in inducing immune responses during parasitic infection and driving the inflammatory responses against invading parasites [17]. However, little is known about the regulation of NLRs and NLR-related inflammasome activation in *T. gondii*-infected human small intestinal epithelial (FHs 74 Int) cells. Therefore, this study evaluated the expression profiles of NLRs, inflammasome components, and caspase-cleaved ILs and investigated the mechanisms of some popular inflammasomes’ activation in *T. gondii*-infected FHs 74 Int cells.

## Methods

### Cell culture

A non-transformed human fetal small intestinal epithelial cell line (FHs 74 Int cells) was purchased from ATCC (ATCC, Manassas, VA, USA) and cultured in DMEM with 10% (v/v) heat-inactivated fetal bovine serum (FBS), an antibiotic–antimycotic solution, and 30 ng/ml human epidermal growth factor (all from Gibco, Grand Island, NY, USA) at 37 °C in a humidified atmosphere at 5% (v/v) CO_2_. The medium was changed every 2–3 days.

### Maintenance of *T. gondii*

Tachyzoites of the *T. gondii* RFP-RH or RH strain were maintained as described previously [16]. Briefly, human retinal pigment epithelial cells (ARPE-19 cells) (ATCC) were cultured in a 1:1 (v/v) mixture of DMEM/F12 supplemented with 10% (v/v) FBS and an antibiotic–antimycotic solution (all from Gibco). ARPE-19 cells were infected with *T. gondii* at a multiplicity of infection (MOI) of 5 for 2–3 days. After spontaneous host cell rupture, parasites and cellular debris were pelleted by centrifugation and washed in cold PBS. The final pellet was resuspended and passed through a 26-gauge needle fitted with a 5.0 μm pore-sized filter (Millipore, Billerica, MA, USA).

### Reverse transcription polymerase chain reaction (RT-PCR)

Total RNA was extracted using TRIzol Reagent (Invitrogen, Carlsbad, CA, USA), and RNA was transcribed into cDNA using M-MLV reverse transcriptase (Invitrogen) as described by the manufacturer. Polymerase chain reaction (PCR) was performed with TaKaRa Ex Taq (Takara Bio, Shiga, Japan) in reactions containing 33.75 μl distilled water, 5 μl 10 × Ex Taq buffer, 4 μl dNTP mixture (2.5 mM each), 2 μl of each primer, 0.25 μl of TaKaRa Ex Taq, and 3 μl of template cDNA to total 50 μl. PCR amplification conditions were an initial denaturation at 95 °C for 5 min, followed by 35 cycles of 95 °C for 30 s, an annealing 60 °C for 30 s, and an extension step of 72 °C for 30 s. Finally, PCR was completed with the additional extension step for 10 min. The PCR products were analyzed on 1.5% agarose gel in 0.5 × TBE buffer and visualized using ethidium bromide and a UV transilluminator. The details of primers designed are presented in Table 1.

**Table 1:** Members of the NLR family, inflammasome components and caspase-cleaved interleukins and primers used to investigate their expressions by RT-PCR and qRT-PCR

Family	Name	Synonym	Forward 5′–3’	Reverse 5′–3’	Amplicon length (bp)
NLRC	NOD1	CARD4	ACTGAAAAGCAATCGGGAACT	ACACACAATCTCCGCATCTTC	112
	NOD2	CARD15	GCCTGATGTTGGTCAAGAAGA	GATCCGTGAACCTGAACTTGA	107
	NLRC3	NOD3, CLR16.2	GGAGCCTCACCAGCTTAGATT	AGGCCACCTGGAGATAGAGAG	117
	NLRC4	IPAF, CARD12	GGAAAGTGCAAGGCTCTGAC	TGTCTGCTTCCTGATTGTGC	129
	NLRC5	NOD27, CLR16.1	CACCCTGACCAACATCCTAGA	TCTCTATCTGCCCACAGCCTA	113
NLRP	NLRP1	NALP1, CARD7	ATACGAAGCCTTTGGGGACT	ACAAAGCAGAGACCCGTGTT	148
	NLRP2	NALP2	CACCGAATGGATCTGTCTGA	GTGGTCGTTCTTTCCGTGTT	112
	NLRP3	NALP3, CIAS1	AAAGGAAGTGGACTGCGAGA	TTCAAACGACTCCCTGGAAC	129
	NLRP4	NALP4	CCAACGAGTTTGGCTGACTT	GCTGTCGATGACGAACAAGA	105
	NLRP5	NALP5	CTGGGGAACGAAGGTGTAAA	GCAAGTGCAAGAAAACCACA	122
	NLRP6	NALP6	CTGTTCTGAGCTACTGCGTGAG	AGGCTCTTCTTCTTCTTCTCCTG	100
	NLRP7	NALP7	TAACCCGTAGCACCTGTCATC	GGTCTTCTTCCCAATGAAAGC	101
	NLRP8	NALP8	CGCTGGTGTGCTTTCTACTTC	GGTCGGGTTTGGACATAATCT	130
	NLRP9	NALP9	CTAGCCTCTCCCAGTCTGACAT	GCGATGTCTTCACAAACTTCAC	121
	NLRP10	NALP10	GTCACGGTGGAGGCTCTATTT	CGAGAGTTGTCTTTCCAGTGC	100
	NLRP11	NALP11	GTGTTGCATGTGACGTTTCC	TTTTGTTGCTCCCAATCTCC	157
	NLRP12	NALP12	CGACCTTTACCTGACCAACAA	AGGTCCATCCCAAATAACCAG	114
	NLRP13	NALP13	ATGGTGTGTTGGACCGTATGT	GCCAAATCTACCTCTGCTGT	140
	NLRP14	NALP14	CCGCTTGTACTTGTCTGAAGC	GCCTCCATCTACTGGTGTGAA	122
NLRB	NAIP1	BIRC1, NLRB1	AGTACTTTTTCGACCACCCAGA	TAGTTGGCACCTGTGATTTGTC	135
NLRA	CIITA	MHC2TA, C2TA	CCGACACAGACACCATCAAC	CCTCTGGGAAGGGTCTTTTC	249
NLRX	NLRX1	NOD9	TGGCCTTGTCTCAGCTCTTTA	CACCAGTCCAGAACCATCTTG	121
Infl. comp	Caspase 1	IL1BC	GGGGTACAGCGTAGATGTGAA	CTTCCCGAATACCATGAGACA	137
	Caspase 5	ICH-3	TCTGTTTGCAAGATCCACGA	GTTCTATGGTGGGCATCTGG	223
	Caspase 8	ALPS2B	AGAAGAGGGTCATCCTGGGAGA	TCAGGACTTCCTTCAAGGCTGC	142
	ASC	PYCARD	CTGACGGATGAGCAGTACCA	CAAGTCCTTGCAGGTCCAGT	108
Interleukins	IL-1β	IL1F2	CCACAGACCTTCCAGGAGAA	GTGATCGTACAGGTGCATCG	121
	IL-18	IL1F4	CACCCCGGACCATATTTATT	TCATGTCCTGGGACACTTCTC	205
	IL-33	IL1F11	GGTGACGGTGTTGATGGTAAG	CTGGCAGTGGTTTTTCACACT	121
	IL-37	IL-1F7	CAGCCTCTGCGGAGAAAGGAAGT	GTTTCTCCTTCTTCAGCTGAAGG	120
Control	HPRT-1	HGPRT	GACCAGTCAACAGGGGACAT	CTGCATTGTTTTGCCAGTGT	111

### Immunocytochemistry

FHs 74 Int cells were seeded onto coverslips in 12-well plates at a density of 1 × 10^4^ cells/well and incubated for 24 h. The cells were infected with *T. gondii* at MOI 10 for 0, 4, and 8 h. Subsequently, the cells were washed with Hank's balanced salt solution (HBSS) and fixed with freshly prepared 4% paraformaldehyde for 1 h at room temperature. After washing five times with PBS containing 0.3% Triton X-100 (PBS-T) for 10 min, the cells were incubated with primary antibodies (α-tubulin, cleaved caspase-8, cleaved IL-1β, IL-33) for 2 h at room temperature. The cells were washed to remove excess primary antibody and then incubated with the appropriate fluorescently labeled secondary antibodies (anti-mouse Alexa Fluor 647, anti-rabbit Alexa Fluor 647, anti-mouse Alexa Fluor 488, and anti-rabbit Alexa Fluor 488) for 2 h at room temperature. After mounting with VECTASHIELD HardSet antifade mounting medium with DAPI (Vector Laboratories, Burlingame, CA, USA), fluorescence images were acquired using a confocal microscope (Leica, Wetzlar, German).

### Lactate dehydrogenase (LDH) assay

LDH assay was performed to quantify cytotoxicity. This assay was conducted using the CytoTox 96 Non-Radioactive Cytotoxicity Assay kit (Promega) according to the manufacturer's protocol. Briefly, 1 × 10^4^ cells were seeded into 96-well plates and infected with *T. gondii* MOI 10 for 0, 4, and 8 h in an incubator (5% CO_2_, 90% relative humidity, 37 °C). Next, 50 µl of the supernatant was transferred into a new 96-well plate, and 50 µl of CytoTox 96 reagent was added and incubated for 30 min at room temperature. After incubation, the absorbance of the solution was measured immediately at 490 nm using a microplate reader (TECAN, Männedorf, Switzerland). LDH levels in the media were quantified and compared to control values according to the kit instructions.

### Real-time quantitative reverse transcription polymerase chain reaction (qRT-PCR)

qRT-PCR was performed using Power SYBR^®^ Green PCR Master Mix (Applied Biosystems, Foster City, CA, USA). The primers used in this study are summarized in Table 1. All reactions were performed with an ABI 7500 Fast Real-Time PCR system (Applied Biosystems, Carlsbad, CA, USA) under the following conditions: 95 °C for 30 s, followed by 40 cycles of 95 °C for 15 s and 60 °C for 30 s. Relative gene expression levels were quantified based on the cycle threshold (Ct) values and normalized to the reference gene hypoxanthine phosphoribosyltransferase 1 (HPRT-1). Each sample was measured in triplicate, and the gene expression levels were calculated using the 2^–ΔΔCt^ method.

### Western blotting

FHs 74 Int cells were infected with *T. gondii* at MOI 10 for 0, 4, and 8 h. FHs 74 Int cells were preincubated with the 30 μM of SB203580 (p38 MAPK inhibitor) and SP600125 (JNK1/2 inhibitor) for 2 h and infected with *T. gondii* MOI 10 for a further 8 h. Subsequently, cell lysates were collected and lysed in ice-cold radio-immunoprecipitation assay (RIPA) buffer (Thermo Fisher Scientific, Grand Island, NY, USA). Protein concentrations were determined using the Bradford assay (Bio-Rad, Hercules, CA, USA). Total protein (30 μg) was resolved on 10–12% SDS-PAGE gels and then transferred to PVDF membranes (Merck Millipore, Billerica, MA, USA). The membranes were blocked with 5% nonfat skim milk in TBS containing 0.1% Tween 20 (TBST) for 1 h and incubated with primary antibodies against NLRP1, NLRP3, NLRP6, NLRC4, NAIP1, cleaved caspase-1, ASC, cleaved IL-1β, IL-33, p-p38 MAPK, p38 MAPK, p-ERK1/2, ERK1/2, p-JNK1/2, JNK1/2, TP3, and α-tubulin overnight at 4 °C. Subsequently, the membranes were incubated with HRP-conjugated secondary antibody (Santa Cruz Biotechnology) for 2 h at room temperature. The membrane was soaked with Immobilon Western Chemiluminescent HRP Substrate (Jackson ImmunoResearch Laboratories), and chemiluminescence was detected with a Fusion Solo System (Vilber Lourmat, Collegien, France). Band intensity was quantified using ImageJ software (NIH, Bethesda, MD, USA). The result was normalized to the α-tubulin protein level and expressed as fold changes compared to the control group.

### ELISA

FHs 74 Int cells were infected with *T. gondii* at MOI 10 for 0, 4, and 8 h. The supernatants from the mock- or *T. gondii*-infected FHs 74 Int cells were collected in triplicate, and IL-1β and IL-18 levels were measured using commercially available ELISA kits following the manufacturer’s instructions (R&D System, Minneapolis, MN, USA). The cytokine concentrations in the samples were calculated from standard curves obtained using recombinant cytokines.

### Statistical analysis

All results are presented as the means ± standard deviations (SDs) of at least three independent experiments, unless otherwise indicated. Statistical comparisons were carried out using GraphPad Prism software (GraphPad Software Inc., San Diego, CA, USA), and multiple t-tests was used to determine one-way ANOVA procedures. Differences were considered significant at *P* < 0.05.

## Results

### Expression of NLRs, inflammasome components, and caspase-cleaved ILs in FHs 74 Int cells

**Fig. 1:**
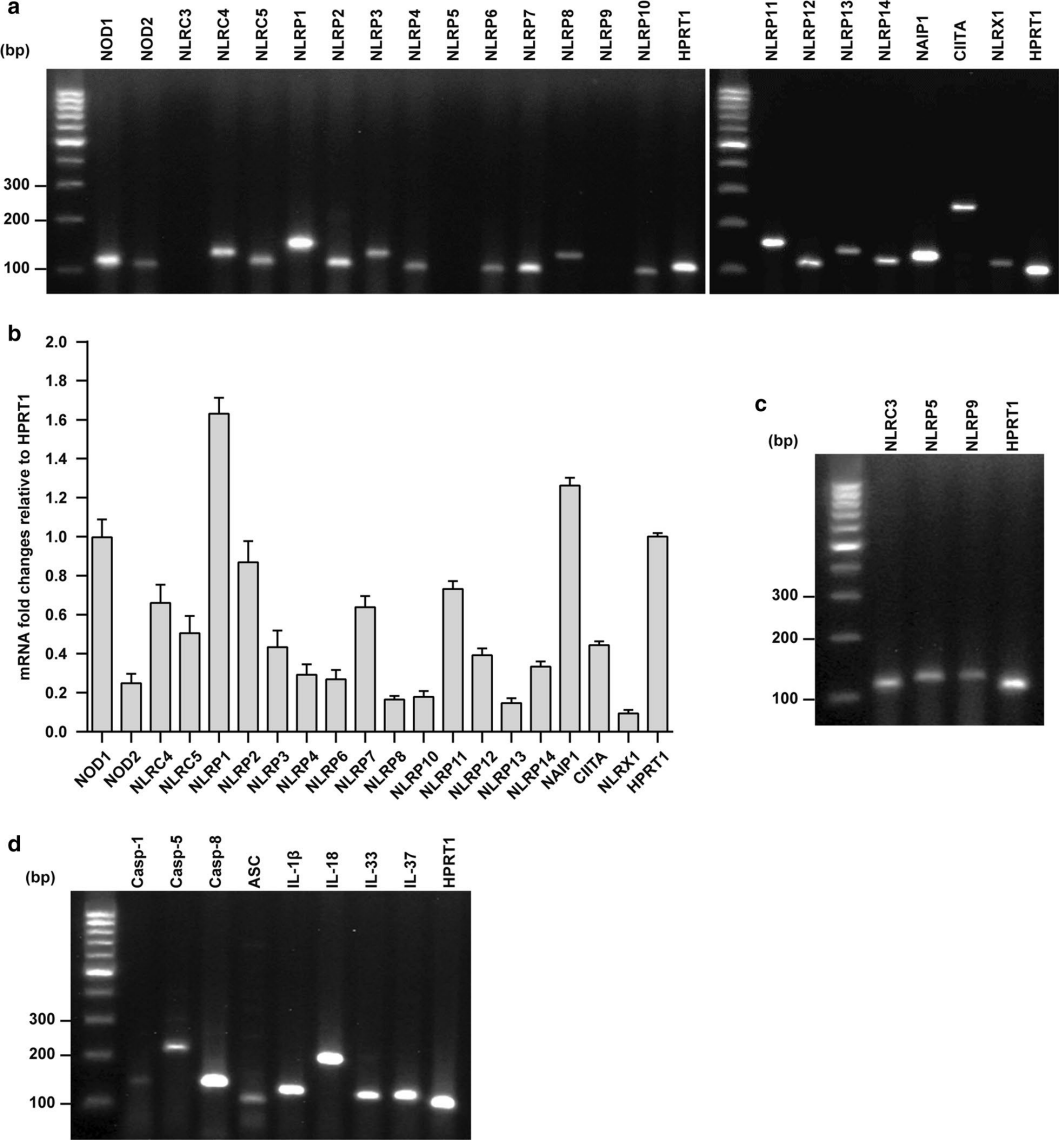
Expression of nucleotide-binding oligomerization domain-like receptors (NLRs), inflammasome components, and caspase-cleaved interleukins (ILs) in human small intestinal epithelial (FHs 74 Int) cells. Total RNA isolated from the untreated cells was examined by polymerase chain reaction (PCR) for mRNA expression of different genes. **a** Expression of NLR mRNAs in FHs 74 Int cells. **b** Expression of the NLR mRNAs in FHs 74 Int cells compared to HPRT-1. **c** Positive controls for primer functionality using human acute monocytic leukemia cell line (THP-1). **d** Inflammasome components and caspase-cleaved ILs expressed in FHs 74 Int cells. Images shown are representatives of five independent experiments

We first checked the expression of the 22 known members of the human NLR family (Table 1) in FHs 74 Int cells using RT-PCR. Our results indicated that the majority of the NLR family members, including NOD1, NOD2, NLRC4, NLRC5, NLRP1, NLRP2, NLRP3, NLRP4, NLRP6, NLRP7, NLRP8, NLRP10, NLRP11, NLRP12, NLRP13, NLRP14, NAIP1, CIITA, and NLRX1 were expressed in these cells under normal conditions (Fig. 1a). After normalization with housekeeping gene HPRT-1, NLRP1 and NOD1 were identified as the most abundantly expressed NLRs in FHs 74 Int cells (Fig. 1b). For primer functionality tests, we used different cell types of human origin that are known to express the respective NLRs and detected the expression of *NLRC3*, *NLRP5*, and *NLRP9* mRNAs (Fig. 1c). Furthermore, we examined the presence of various inflammasome components and ILs cleaved by caspases. Our results revealed that caspase-1, caspase-5, caspase-8, and ASC inflammasome components and IL-1β, IL-18, IL-33, and IL-37 were expressed in the FHs 74 Int cells (Fig. 1d).

### Effects of *T. gondii* infection on cell morphology and cytotoxicity of FHs 74 Int cells

**Fig. 2:**
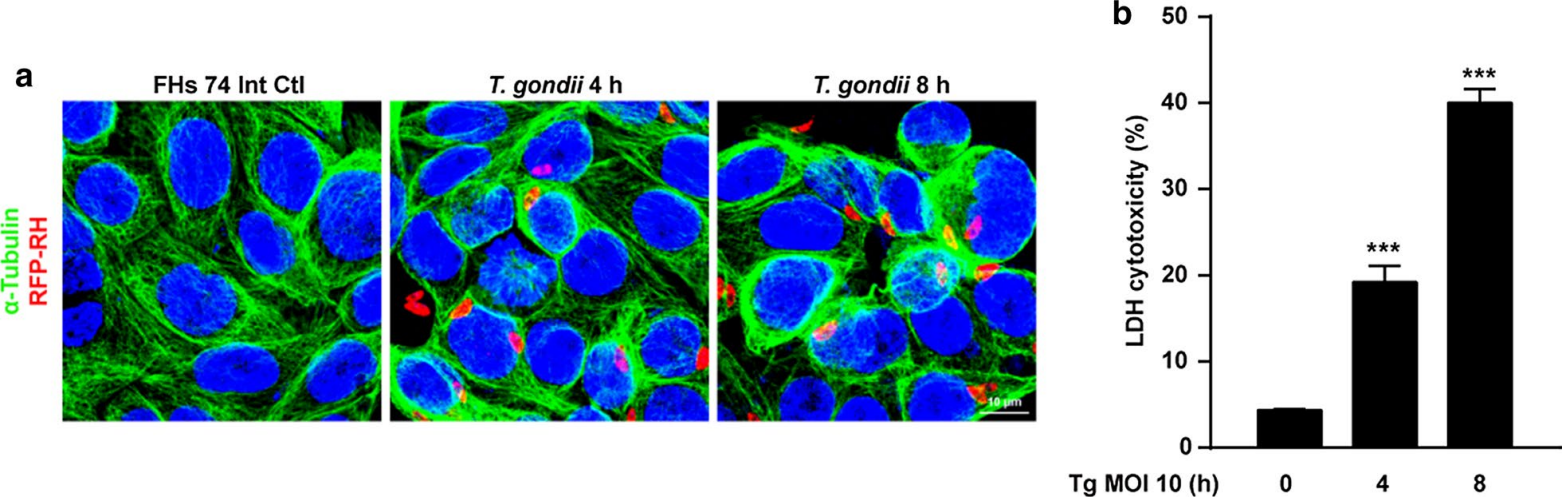
Effects of *T. gondii* infection on cell morphology and cytotoxicity of FHs 74 Int cells. FHs 74 Int cells were infected with RFP-RH *T. gondii* strain at a multiplicity of infection (MOI) of 10 for 0, 4, and 8 h. **a** Cells fixed and stained with α-tubulin (green) and nuclei stained with DAPI (blue) were observed by confocal microscopy. **b** Lactate dehydrogenase level in the media, which is related with cell death, was measured in FHs 74 Int cells infected with *T. gondii* at a MOI of 10 for 0, 4, and 8 h. The percentage of cytotoxicity was calculated. Horizontal lines in each group represent mean ± standard deviation (SD) value of at least three independent experiments. ^***^P < 0.001, as compared to the control group

FHs 74 Int cells were incubated with *T. gondii* at MOI of 10 for various time periods. The integrity of the microtubule network was assessed with immunofluorescence microscopy using α-tubulin antibody and DAPI for staining cellular microtubules and DNA, respectively. As shown in Fig. 2a, the cell nucleus (blue) was wrapped with a well-developed array of hair-like microtubule networks of slim fibrous microtubules (green) in control cells. In contrast, the α-tubulin staining patterns were diffuse and disorganized in *T. gondii*-infected FHs 74 Int cells. The number of *T. gondii*-infected cells and the total number of cells were counted under a fluorescence microscope. The *T. gondii* infection rate significantly increased in an infection time-dependent manner (73.1% at 4 h and 89.5% at 8 h).

Furthermore, to investigate *T. gondii*-induced cytotoxicity of FHs 74 Int cells, the cells were incubated with *T. gondii* at an MOI of 10 for 0, 4, and 8 h. Post-incubation, LDH assay was performed. Release of LDH significantly increased in the *T. gondii*-infected groups compared to that in the mock-infected control group. Cytotoxicities of FHs 74 Int cells infected with *T. gondii* for 0, 4, and 8 h were 4.34 ± 0.15%, 19.21 ± 1.88%, and 40.02 ± 1.57%, respectively (Fig. 2b). These data indicate that *T. gondii* infection induces morphological disorganization and cytotoxicity in FHs 74 Int cells in an infection time-dependent manner.

### Transcriptional regulation of NLRs in FHs 74 Int cells

**Fig. 3:**
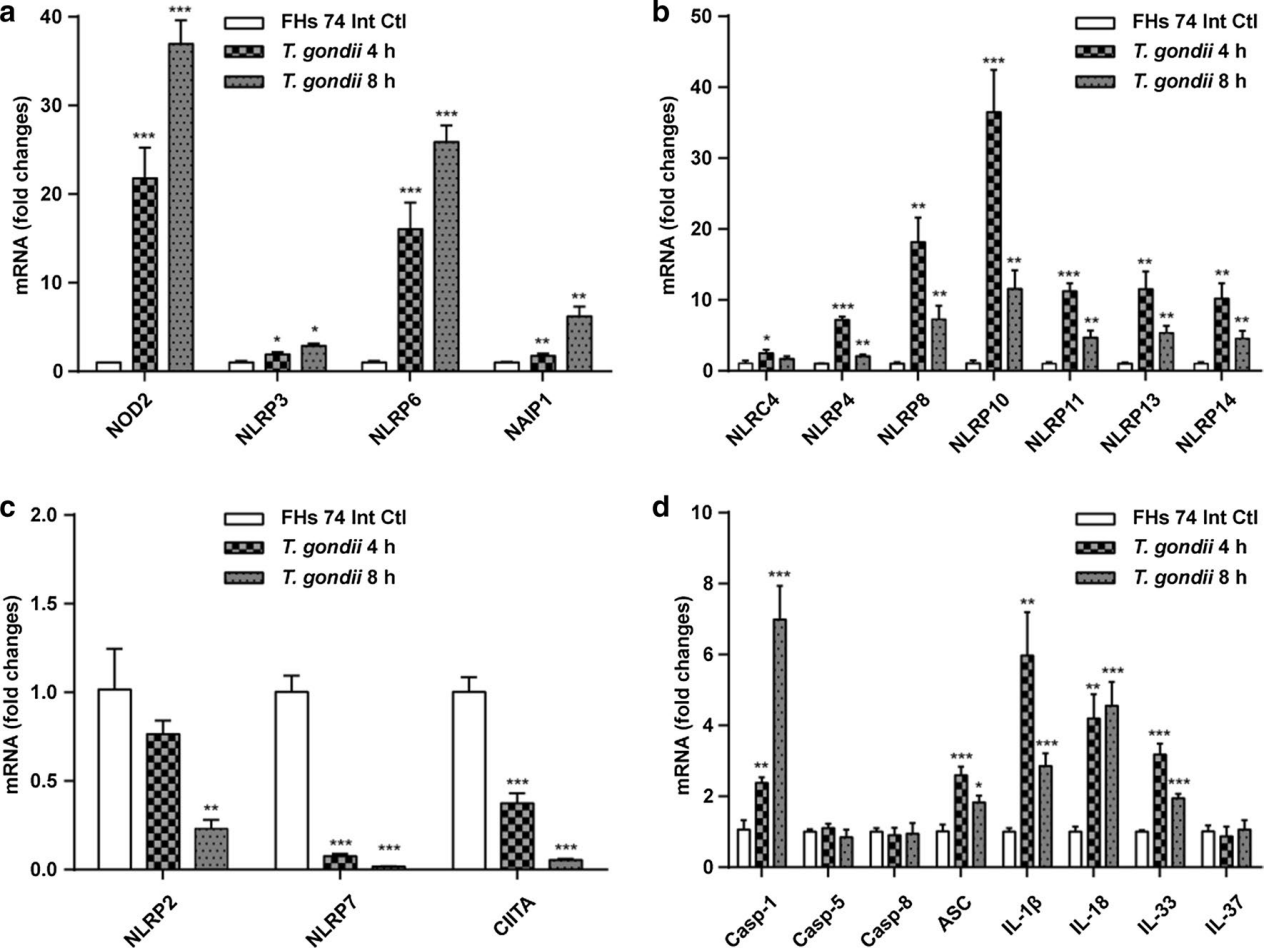
Induction of NLRs, inflammasome components, and caspase-cleaved ILs in response to *T. gondii* infection in FHs 74 Int cells. FHs 74 Int cells were infected with RFP-RH *T. gondii* strain at a MOI of 10 for 0, 4, and 8 h. **a**–**d** Expression of the NLR mRNAs, inflammasome components, and caspase-cleaved ILs in FHs 74 Int cells. Each PCR was carried out with three parallels. ^*^P < 0.05, ^**^P < 0.01, ^***^P < 0.001 compared to control

Next, we aimed to investigate the expression of the identified NLRs in response to *T. gondii* infection for 4 or 8 h. Real-time qRT-PCR revealed that *T. gondii* infection induces a significant time-dependent increase in the expression of *NOD2*, *NLRP3*, *NLRP6*, and *NAIP1* mRNAs (Fig. 3a). Interestingly, *T. gondii* infection upregulated the expression of *NLRC4*, *NLRP4*, *NLRP8*, *NLRP10*, *NLRP11*, *NLRP13*, and *NLRP14* mRNAs at both 4 and 8 h post-infection, but *NLRP4*, *NLRP8*, *NLRP10*, and *NLRP11* mRNAs were significantly downregulated at 8 h post-infection compared to that at 4 h post-infection (Fig. 3b). In contrast, *T. gondii* infection induced a time-dependent significant decrease in the expression of *NLRP2*, *NLRP7*, and *CIITA* mRNAs (Fig. 3c). No significant changes in the expression of *NOD1*, *NLRC3*, *NLRC5*, *NLRP1*, *NLRP9*, *NLRP12*, and *NLRX1* mRNAs were noted as a result of *T. gondii* infection (data not shown). Neither normal nor *T. gondii*-infected FHs 74 Int cells expressed *NLRP5* mRNA. While *T. gondii* infection increased the expression of mRNAs encoding caspase-1, ASC, IL-1β, IL-18, and IL-33, it had no effect on the expression of mRNAs encoding caspase-5, caspase-8, and IL-37 (Fig. 3d). These results clearly indicate that *T. gondii* infection activates NLRs, but their expression patterns vary in FHs 74 Int cells.

### *T. gondii* infection induced NLRP3, NLRP6, and NLRC4 inflammasome components in FHs 74 Int cells

**Fig. 4:**
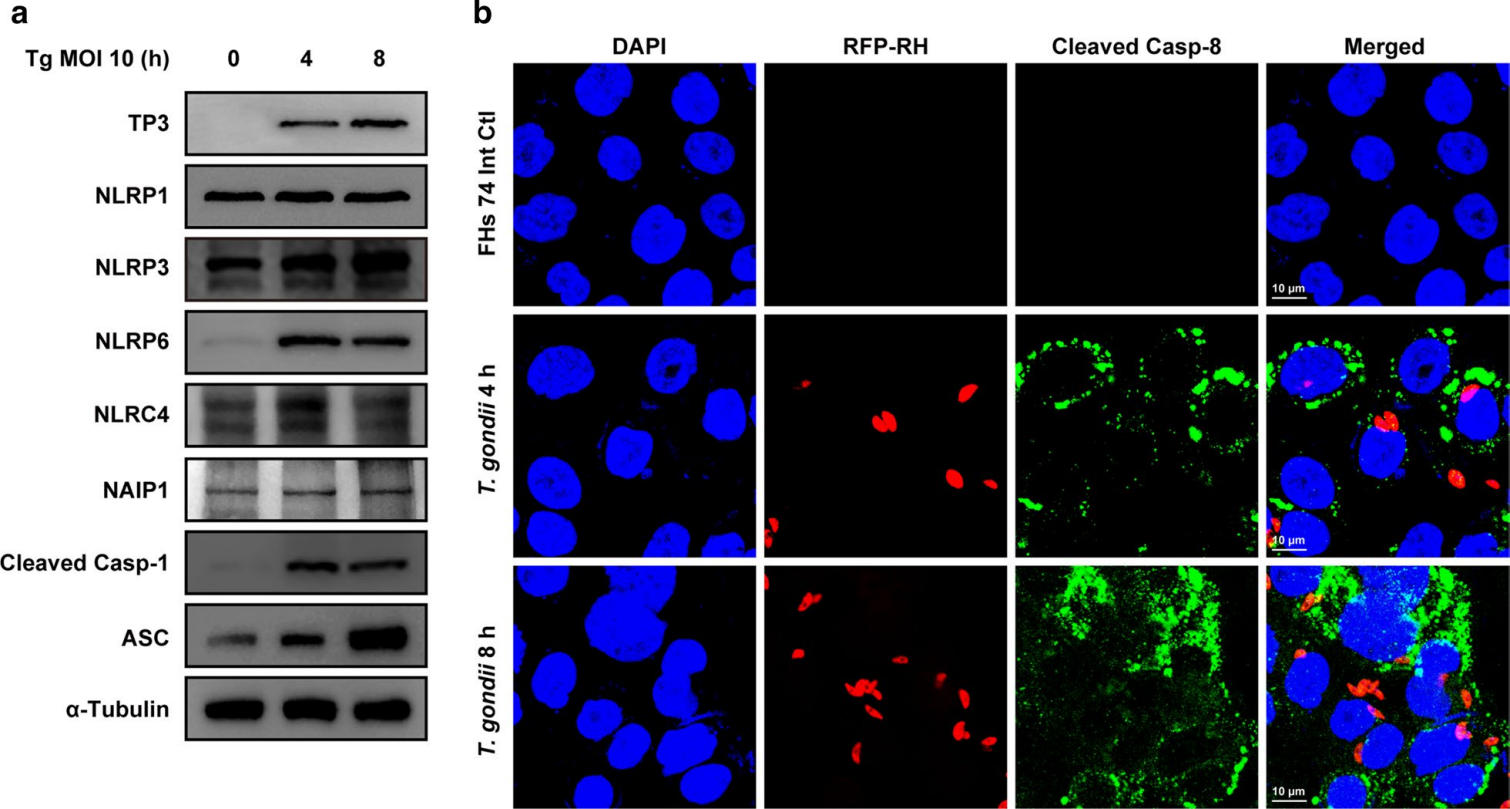
Expression of NLR family members, NLRP1, NLRP3, NLRP6, and NLRC4 inflammasome components in response to *T. gondii* infection in human small intestinal epithelial (FHs 74 Int) cells. **a** FHs 74 Int cells were infected with *T. gondii* at a MOI of 10 for 0, 4, and 8 h. Expression of NLRP1, NLRP3, NLRP6, and NLRC4 inflammasome component proteins were detected by western blot analysis; α-tubulin was used as the loading control. **b** FHs 74 Int cells were fixed and probed against cleaved caspase-8 (green). The cells were counterstained with 4′,6-diamidino-2-phenylindole (blue) and visualized using confocal microscopy. All data shown are representative of three independent experiments with similar results

Until now, the most commonly studied inflammasomes in protozoan parasites were NLRP1, NLRP3, and NLRC4 [15]. Thus, we further investigated the protein levels of NLRP1, NLRP3, NLRP6, and NLRC4 inflammasome components in response to *T. gondii* infection. *T. gondii* infection time-dependently induced NLRP3 and ASC protein expression, adequately induced NLRP6 and cleaved caspase-1 expression, and moderately induced NLRC4 expression at 4 h post-infection. However, expression levels for NLRP1 and NAIP1 proteins remained unchanged in response to *T. gondii* infection (Fig. 4a). Confocal microscopy revealed that the expression of cleaved caspase-8 was higher in *T. gondii*-infected FHs 74 Int cells compared to that in mock-infected control FHs 74 Int cells (Fig. 4b). These results indicate that *T. gondii* infection induces NLRP3, NLRP6, and NLRC4 inflammasome activation in FHs 74 Int cells.

### *T. gondii* infection upregulates ILs expression and release in FHs 74 Int cells

**Fig. 5:**
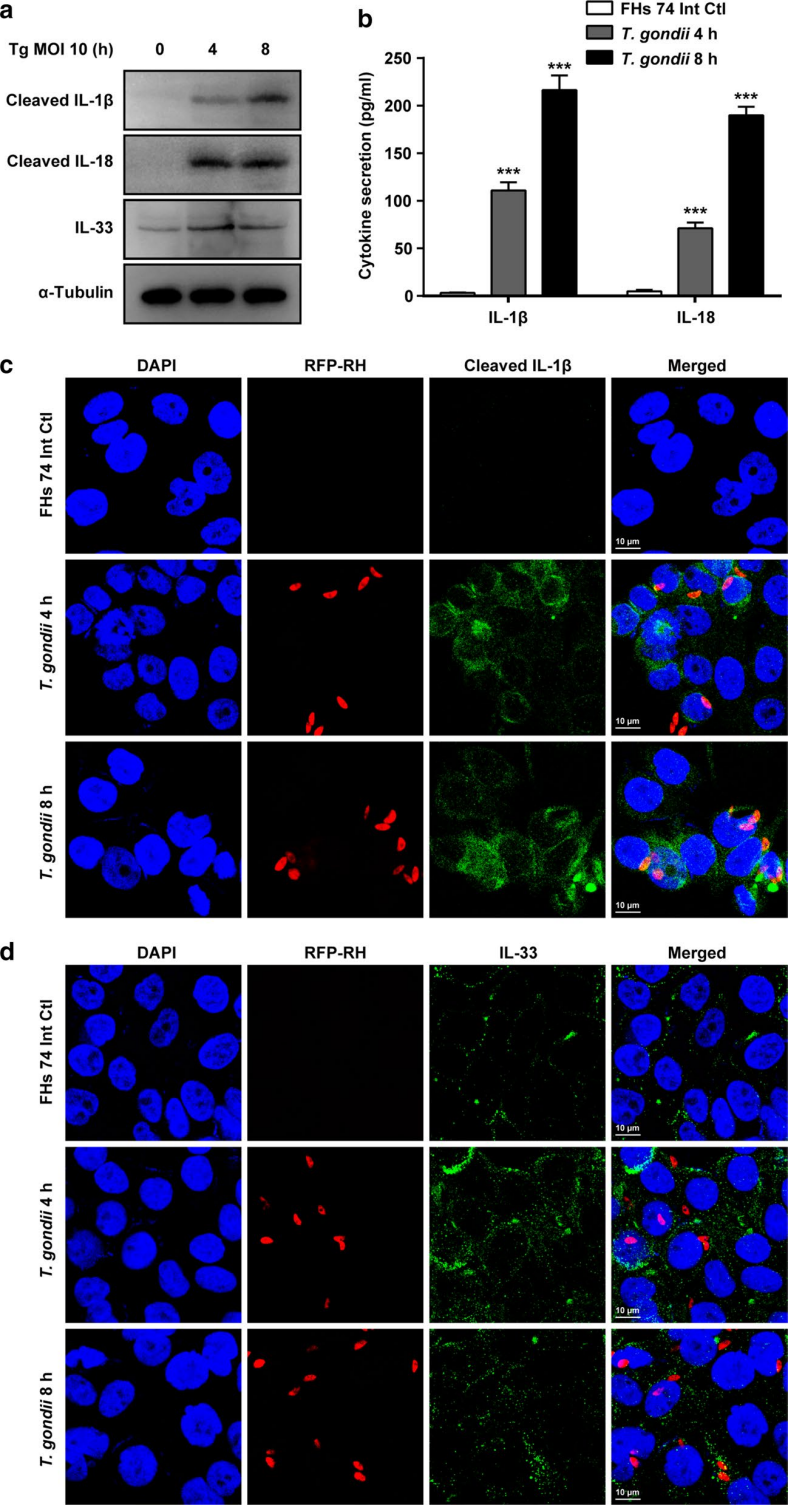
Caspase-cleaved ILs in response to *T. gondii* infection in FHs 74 Int cells. **a** FHs 74 Int cells were infected with *T. gondii* at a MOI of 10 for 0, 4, and 8 h. Protein levels of ILs were detected by western blot analysis; α-tubulin was used as the loading control. **b** IL-1β and IL-18 protein levels in the culture medium of FHs 74 Int cells after *T. gondii* infection were measured by ELISA. Graphs show quantified results in pg/ml as mean ± standard error. ^***^P < 0.001 compared to control. **c**, **d** FHs 74 Int cells were fixed and probed against cleaved IL-1β and IL-33. The cells were counterstained with 4′,6-diamidino-2-phenylindole (blue) and visualized using confocal microscopy. All data shown are representative of three independent experiments with similar results

NLRs are a large group of cytosolic sensors whose main function is to modulate the expression of proinflammatory cytokines [8, 10–14]. Hence, we evaluated the protein expression levels of IL-1β, IL-18, IL-33, and IL-37 in *T. gondii*-infected FHs 74 Int cells. Western blot analysis results revealed upregulated expression of cleaved IL-1β, cleaved IL-18, and IL-33 proteins in the *T*. *gondii*-infected FHs 74 Int cell lysates (Fig. 5a). The concentrations of released IL-1β and IL-18 were measured in the *T. gondii*-infected FHs 74 Int cell culture medium. *T. gondii* infection induced a robust increase in the amount of active IL-1β and IL-18 in the culture medium (Fig. 5b). Confocal microscopy detected similar expression levels of cleaved IL-1β and IL-33. In control cells, cleaved IL-1β levels were non-detectable, while in *T. gondii*-infected cells, activated IL-1β and IL-33 increased significantly (Fig. 5c, d). These results indicate that *T. gondii* induces IL-1β, IL-18, and IL-33 production in FHs 74 Int cells.

### *T. gondii*-induced NLRP3 inflammasome activation is strongly associated with the phosphorylation of p38 MAPK

**Fig. 6:**
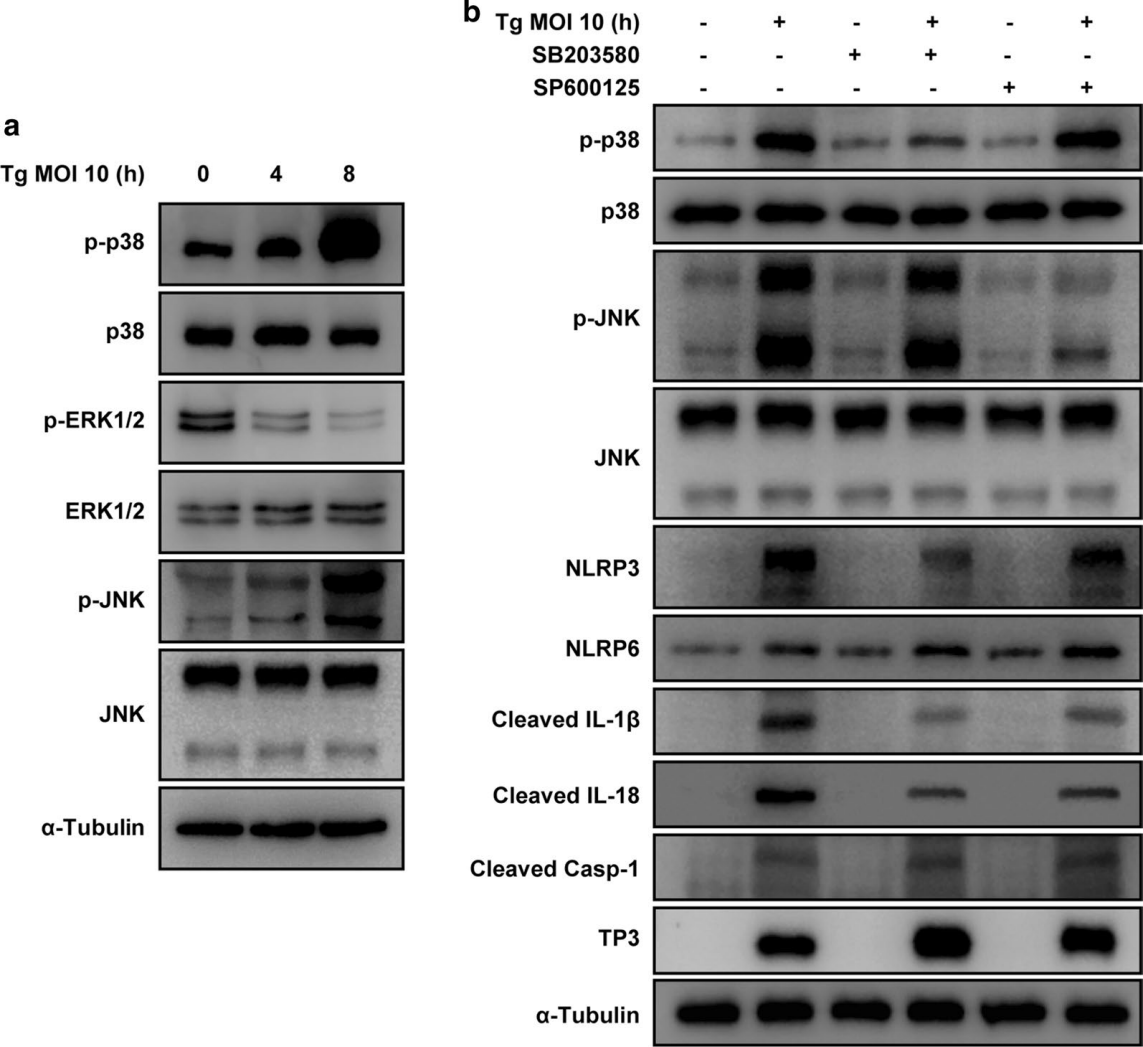
Signaling pathways involved in the regulation of NLRP3 and NLRP6 inflammasome components. **a** FHs 74 Int cells were infected with *T. gondii* at a MOI of 10 for the indicated time periods. Activation of mitogen-activated protein kinase (MAPK) subsets was evaluated by western blotting. **b** FHs 74 Int cells were preincubated with the 30 μM of SB203580 (p38 MAPK inhibitor) and SP600125 (JNK inhibitor), respectively, for 2 h and then infected with *T. gondii* at a MOI of 10 for 8 h. The indicated protein levels were then evaluated by western blot. Anti-α-tubulin was used as the internal control. Similar results were obtained in three independent experiments

Studies have reported that the mitogen-activated protein kinase (MAPK) pathway is associated with inflammasome activation [18, 19]. Hence, we investigated the involvement of the MAPK pathway in *T. gondii*-induced NLRP3 and NLRP6 inflammasome activation. As shown in Fig. 6a,* T. gondii* infection increased the levels of phosphorylated p38 MAPK and JNK1/2; however, the level of phosphorylated ERK1/2 decreased compared with that in the control cells. There were no significant changes in the total protein levels of ERK1/2, p38 MAPK, and JNK1/2 after *T. gondii* infection. However, pretreatment with SB203580 (p38 inhibitor) and SP600125 (JNK inhibitor) significantly decreased the phosphorylation of p38 MAPK and JNK1/2 in *T. gondii*-infected cells compared with that in inhibitor-untreated *T. gondii*-infected cells. Interestingly, pretreatment with SB203580 significantly downregulated *T. gondii* infection-induced NLRP3 expression; however, the effects of SP600125 pretreatment were considerably less than those of SB203580 pretreatment. Pretreatment with SB203580 and SP600125 had no effect on the regulation of *T. gondii*-induced NLRP6 activation. SB203580 and SP600125 pretreatment significantly attenuated *T. gondii*-induced elevation in the cleaved IL-1β and cleaved IL-18 levels. Furthermore, pretreatment with SB203580 and SP600125 considerably upregulated the *T. gondii* antibody (TP3) level in *T. gondii*-infected FHs 74 Int cells compared with that in untreated *T. gondii*-infected cells (Fig. 6b).

**Fig. 7:**
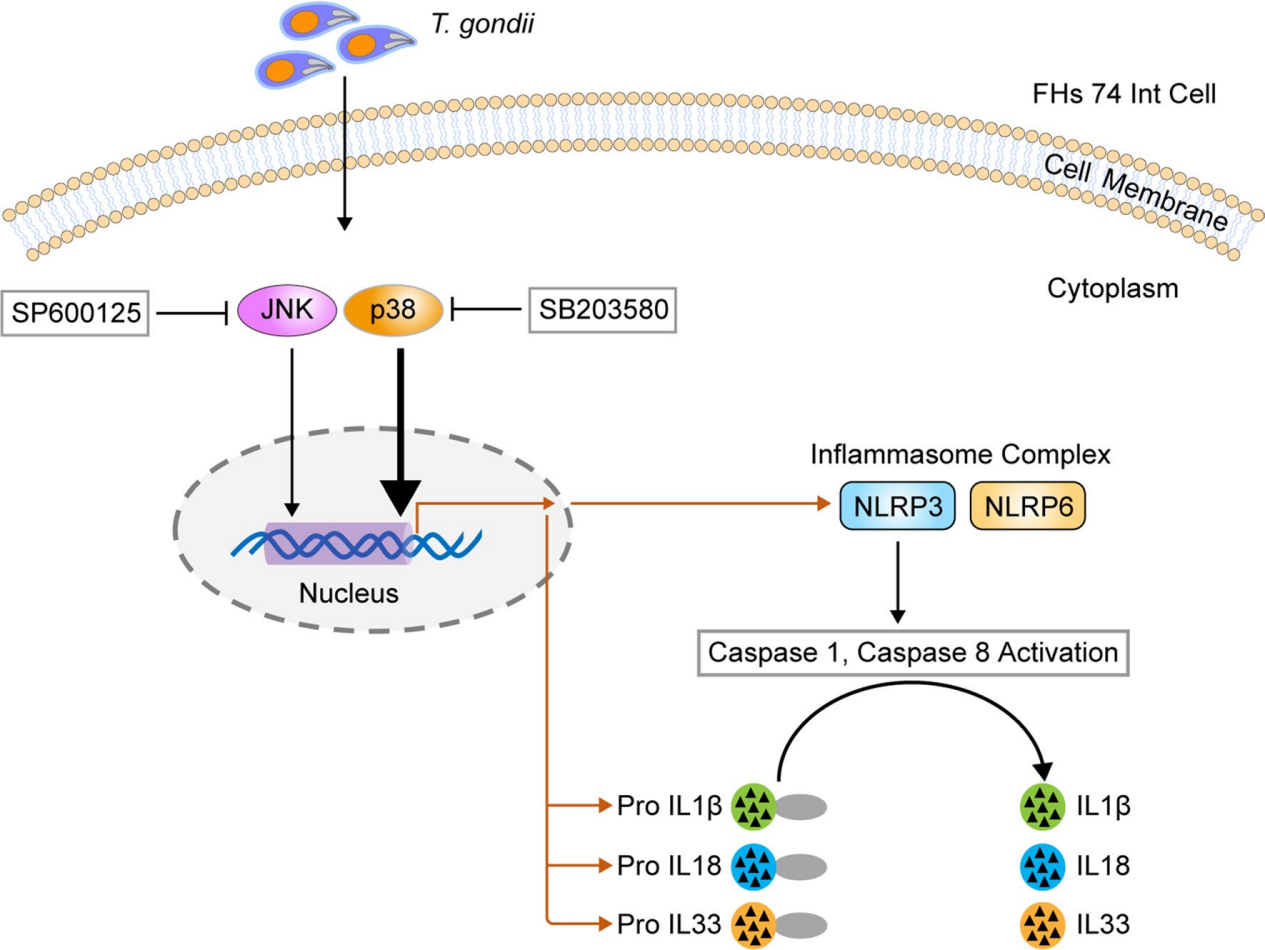
Schematic model of NLRP3 inflammasome activation in *T. gondii*-infected FHs 74 Int cells. *T. gondii* activates the p38 MAPK pathway in small intestinal epithelial cells, subsequently upregulating protein expression and promoting the formation of the NLRP3 inflammasome. The NLRP3 inflammasome cleaves pro-IL-1β, pro-IL-18 and pro-IL-33 to become active IL-1β, IL-18, and IL-33, which ultimately induces cytotoxicity of FHs 74 Int cells

Overall, these results indicate that the p38 MAPK pathway is strongly associated with *T. gondii-*induced NLRP3 inflammasome production in *T. gondii*-infected small intestinal epithelial FHs 74 Int cells, although the p38 MAPK and JNK1/2 pathways are involved in NLRP3 activation. However, NLRP6 inflammasome activation was not related to the MAPK pathway in FHs 74 Int cells (Fig. 7).

## Discussion

This study revealed that members of the NLRs, inflammasome components, and caspase-cleaved ILs are expressed differently in the FHs Int 74 cells under normal and *T. gondii*-infected conditions; however NLRC3, NLRP5, and NLRP9 were not expressed. The most abundantly expressed NLRs were NLRP1 and NOD1. *T. gondii* infection induced cytotoxicity in FHs 74 Int cells in an infection time-dependent manner. In addition, the expression of *NOD2*, *NLRP3*, *NLRP6*, and *NAIP1* mRNAs significantly increased in *T. gondii-*infected cells, while that of *NLRP2*, *NLRP7*, and *CIITA* mRNAs decreased. *T. gondii* infection also induced NLRP3, NLRP6, and NLRC4 inflammasome activation and significantly produced IL-1β, IL-18, and IL-33 in FHs 74 Int cells. NLRP3 inflammasome activation was strongly associated with the p38 MAPK pathway in *T. gondii*-infected cells; however, no relationship was revealed between NLRP6 inflammasome activation and MAPK pathway.

Expression of the NLR family as a pattern recognition receptor is cell specific; however, little was known about the regulation of NLRs and their activation mechanisms in intestinal epithelial cells. In this study, the expression patterns of the NLRs, inflammasome components, and caspase-cleaved ILs were varied in the FHs Int 74 cells under normal and *T. gondii*-infected conditions. Although a previous study reported the expression patterns of NLRs in cerebral endothelial cells [8], this is the first study about the regulation of whole NLRs in the human intestinal cells after *T. gondii* infection. NLRs can be regulated by a wide range of cellular damages, including oxidative stress and inflammatory stimuli [5, 7]. Thus, upon evaluating the cellular changes in *T. gondii*-infected FHs 74 Int cells by immunofluorescence and LDH assay, we observed disorganized staining patterns for α-tubulin and significantly increased release of LDH in proportion to time. These results clearly indicated that *T. gondii* infection induces cellular damage and cytotoxicity in FHs 74 Int cells by activation of inflammasome-related components. Similar phenomena were observed for *Schistosoma mansoni* infection, wherein elicited host immune responses resulted in mitochondrial damage, generation of high levels of reactive oxygen species (ROS), and activation of apoptosis through interaction with host inflammasomes [20]. In addition, *Neospora caninum*-induced NADPH-dependent ROS generation plays an important role in NLRP3 inflammasome activation [21].

The present investigation in *T. gondii*-infected FHs 74 Int cells revealed that *T. gondii* infection induces the expression of NLRP3, ASC, NLRP6, NLRC4, cleaved caspase-1, and cleaved caspase-8. In addition, increased production of ILs such as IL-1β, IL-18, and IL-33 was observed in *T. gondii-*infected FHs 74 Int cells. These findings suggest that *T. gondii* infection induces the activation of NLRP3, NLRP6, and NRC4 inflammasomes via the recruitment of ASC and caspases and production of proinflammatory cytokines in FHs 74 Int cells. These findings are partially consistent with those of a previous study, that is, NLRP3, ASC, caspase-1, and IL-1β were detected in *T. gondii*-infected mice [11]. The findings are also consistent with those of our previous study, that is, *T. gondii* infection of THP-1 macrophages increased the production of IL-1β and inflammasome sensors, including NLRP1, NLRP3, NLRC4, NLRP6, NLRP8, NLRP13, AIM2, and NAIP, in a time-dependent manner [12]. Compared with the findings of previous studies, the expression patterns of inflammasome components in this study were slightly different, and this can be attributed to the cell type and culture conditions.

MAPKs, highly conserved in all eukaryotes, control a variety of cellular processes, including cell differentiation, proliferation, survival, and stress responses. It has been reported that the MAPK pathway is associated with inflammasome activation [18, 20, 22]. In the present study, we evaluated the roles of the MAPK signaling pathways in NLRP3 and NLRP6 inflammasome activation in *T. gondii*-infected FHs 74 Int cells by pretreatment with SB203580 and SP600125, inhibitors of p38 MAPK and JNK, respectively. While SB203580 pretreatment significantly downregulated *T. gondii*-induced expression of NLRP3, cleaved IL-1β, and cleaved IL-18, the effects of SP600125 pretreatment were less than those of SB203580 pretreatment. However, NLRP6 activation was not affected by pretreatment with SB203580 or SP600125 in *T. gondii*-infected FHs 74 Int cells. These findings suggest that p38 MAPK signaling is a more important factor than JNK1/2 signaling, although both signaling pathways were involved with NLPR3 activation in *T. gondii-*infected FHs 74 Int cells. However, NLRP6 inflammasome activation had no correlation with the MAPK pathway.

We also compared the parasite levels in *T. gondii*-infected FHs 74 Int cells after pretreatment with SB203580 or SP600125 to evaluate the host cell environment for parasite proliferation. Both inhibitors apparently increased the parasite proliferation compared with that in the untreated group of *T. gondii*-infected FHs 74 Int cells. However, SB203580 pretreatment induced higher TP3 expression than SP600125 pretreatment in *T. gondii-*infected FHs 74 Int cells, and this was opposite to the expression pattern of NLRP3 inflammasome and IL-1β and IL-18 proteins after pretreatment with SB203580 and SP600125. These data suggest that NLRP3 inflammasome is important for restricting *T. gondii* proliferation in *T. gondii*-infected FHs 74 Int cells, and the p38 MAPK pathway is a more critical factor than the JNK1/2 pathway in the regulation of *T. gondii*-induced NLRP3 inflammasome production. Our results are consistent with those of previous studies, that is, *T. gondii* triggers NLRP3 or NLRP1, which leads to parasite restriction and resistance to toxoplasmosis [11, 16], and p38 MAPK is important for the regulation of NLRP1 or NLRP3 inflammasome activation and IL-1β secretion [18, 19]. However, our results were contrary to those of Wei et al. [23], who reported that the addition of SB203580 after *T. gondii* infection significantly inhibited *T. gondii* tachyzoite replication in fibroblasts. These different effects on *T. gondii* growth may be a result of differences in cell type, treatment method, time of inhibitor treatment, *T. gondii* infection time, and host cell microenvironments.

## Conclusion

We demonstrated the regulation of NLRs and NLR-related inflammasome activation in *T. gondii*-infected human small intestinal epithelial (FHs 74 Int) cells. *T. gondii* infection induced the expression of NLRs, inflammasome components, and caspase-cleaved ILs in the FHs Int 74 cells, but their expression patterns were varied. NLRP3 inflammasome activation was strongly associated with the p38 MAPK pathway; however, NLRP6 inflammasome activation had no correlation with the MAPK pathway. We believe that the study findings will contribute to the understanding of mucosal and innate immune responses induced by NLRs and inflammasomes in *T. gondii*-infected FHs Int 74 cells.

## Data Availability

All data generated or analyzed during the present study are included in this published article.

## Abbreviations

BSA: Bovine serum albumin
DAPI: 4′,6-Diamidino-2-phenylindole
ELISA: Enzyme-linked immunosorbent assay
FBS: Fetal bovine serum
HPRT-1: Hypoxanthine phosphoribosyltransferase 1
ILs: Interleukins
LDH: Lactate dehydrogenase
MAPK: Mitogen-activated protein kinase
MOI: Multiplicities of infection
NLRs: Nucleotide-binding oligomerization domain (NOD)-like receptors
NOD: Nucleotide-binding oligomerization domain
PBS: Phosphate buffer saline
PCR: Polymerase chain reaction
qPCR: Quantitative polymerase chain reaction
SD: Standard deviation
